# Heterogeneity in Cancer

**DOI:** 10.3390/cancers17030441

**Published:** 2025-01-28

**Authors:** William J. MacDonald, Connor Purcell, Maximilian Pinho-Schwermann, Nolan M. Stubbs, Praveen R. Srinivasan, Wafik S. El-Deiry

**Affiliations:** 1 Laboratory of Translational Oncology and Experimental Cancer Therapeutics, The Warren Alpert Medical School, Brown University, Providence, RI 02903, USA; william_macdonald@alumni.brown.edu (W.J.M.); maximilian_pinho-schwermann@brown.edu (M.P.-S.); nstubbs@msm.edu (N.M.S.);; 2 Department of Pathology and Laboratory Medicine, The Warren Alpert Medical School, Brown University, Providence, RI 02903, USA; 3 Legorreta Cancer Center at Brown University, The Warren Alpert Medical School, Brown University, Providence, RI 02903, USA; 4 Morehouse School of Medicine, Atlanta, GA 30310, USA; 5 The Joint Program in Cancer Biology, Brown University and Brown University Health, Providence, RI 02903, USA; 6 Hematology-Oncology Division, Department of Medicine, Rhode Island Hospital, Brown University, Providence, RI 02903, USA

**Keywords:** drug resistance, cancer heterogeneity, epigenetics

## Abstract

Cancer heterogeneity is a major challenge in oncology, complicating diagnosis, prognostication, and treatment. The clinical heterogeneity of cancer, which leads to differential treatment outcomes between patients with histopathologically similar cancers, is attributable to molecular diversity manifesting through genetic, epigenetic, transcriptomic, microenvironmental, and host biology differences. Heterogeneity is observed between patients, individual metastases, and within individual lesions. This review discusses clinical implications of heterogeneity, emphasizing need for personalized approaches to overcome challenges posed by cancer’s diverse presentations. Understanding of emerging molecular diagnostic and analytical techniques can provide a view into the multidimensional complexity of cancer heterogeneity. With over 90% of cancer-related deaths associated with metastasis, we additionally explore the role heterogeneity plays in treatment resistance and recurrence of metastatic lesions. Molecular insights from next-generation sequencing, single-cell transcriptomics, liquid biopsy technology, and artificial intelligence will facilitate the development of combination therapy regimens that can potentially induce lasting and even curative treatment outcomes.

## Simple Summary

Cancer is characterized by variability at several levels. Individual cells within a tumor exhibit genetic and non-genetic differences, tumors within a patient exhibit different characteristics, and patients themselves have different responses to therapeutics and clinical outcomes. The heterogeneous nature of cancer complicates the ability to predict patient outcomes and the delivery of curative treatment. The present study explores the ways in which the molecular heterogeneity of cancer impacts clinical outcomes, with a unique focus on how emerging technologies may be leveraged to improve patient care.

## 1. Introduction

**Figure 1:**
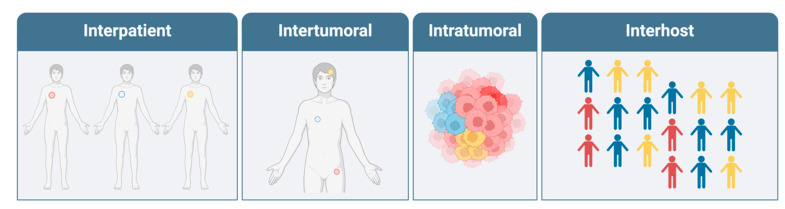
Layers of heterogeneity in cancer.

Cancer, a complex and multifaceted disease, is clinically characterized by an inherent heterogeneity. Heterogeneity underlies the diverse clinical presentations and therapeutic responses observed among patients with ostensibly similar cancers, making personalized medicine an essential yet challenging goal. This variability manifests at multiple levels, including genetic, epigenetic, transcriptomic, phenotypic, and microenvironmental differences within and between patients and individual lesions. This review aims to provide a wide-ranging overview of the molecular mechanisms that drive interpatient, intertumoral, intratumoral, and host heterogeneity (Figure 1).

Interpatient heterogeneity refers to genotypic and phenotypic diversity in tumors across different patients with histopathologically similar cancers originating from the same sites (e.g., head and neck squamous cell carcinoma, non-small cell lung cancer, clear cell renal cell cancer, etc.). Although many of these cancers can be categorized into major histological categories, differences in progression and treatment response across patients with seemingly similar cancers indicate that significant molecular diversity is a major challenge to treatment.

Intratumoral heterogeneity refers to the genetic and phenotypic diversity within a single tumor, which is driven by the continuous evolution of multiple clonal populations under selective pressure. This evolution results in subclones of cancer cells with distinct molecular alterations, be that at a genotypic or phenotypic level. This reservoir of genetic and phenotypic diversity within a single tumor contributes greatly to treatment resistance and disease recurrence.

Intertumoral heterogeneity encompasses the differences between tumors at different sites within a single patient, comparing primary lesions with metastases or metastases among each other. Intertumoral heterogeneity is influenced by factors such as the tissue of origin, metastatic colonization, vascular access, and the surrounding tumor microenvironment of different metastatic sites.

Lastly, we will discuss the concept of host heterogeneity, which encompasses the noncancerous differences that foster the variable responses observed between patients, even those with similar molecular subtypes of cancers. Differences in genetics, metabolism, environmental factors, and lifestyle factors constitute host heterogeneity and can have drastic effects on treatment and cancer outcomes.

Understanding cancer heterogeneity is critical for developing more effective diagnostic tools and therapeutic strategies and for administering existing therapeutic regimens effectively. Advances in high-throughput next-generation sequencing, single-cell resolution sequencing, liquid biopsy technology, and computational modeling have provided deeper insights into the diversity and complexity of cancer. These technologies have revealed the clonal architecture of tumors, the dynamics of clonal evolution, and the impact of the tumor microenvironment on influencing the evolution of clonal populations, all of which are heterogeneity-related concepts that drive cancer progression and therapy resistance.

We will discuss the origins and implications of interpatient, intertumoral, intratumoral, and host heterogeneity, highlighting the latest methodological advancements and their contributions to cancer research. With over 90% of cancer-related deaths associated with metastasis, we will additionally explore the clinical implications of specific forms of heterogeneity on treatment resistance and the recurrence of metastases [1]. This discussion will investigate the need for personalized approaches in cancer treatment and the potential for future therapeutics to overcome the challenges posed by the heterogeneity of cancer.

## 2. Cellular and Molecular Heterogeneity

Prior to the advance of molecular and sequencing technologies, cancers could only be stratified into histopathological subtypes that characterized tumors based on cellular histology and the stage of cancer progression. However, patients with histologically similar cancer from the same tissue of origin have long been documented to experience disparate treatment responses and outcomes [2,3]. The introduction of clinical immunohistochemistry in the 1980s and the advent of sequencing technologies permitted insight into the molecular dimension of cancer, helping foster a scientific understanding that more closely correlates with the observed clinical realities of cancer care [4,5]. A comprehension and cataloging of these molecular subtypes also opened a window to the exploitation of the molecular dependencies of cancer, ushering in the era of targeted therapies.

## 3. Interpatient Heterogeneity

A prominent example of interpatient heterogeneity is breast cancer. A landmark paper first categorized breast cancer into four distinct subtypes based on molecular differences [6]. These include luminal A and luminal B, which are hormone receptor (HR) overexpressing cancers, and basal-like and human epidermal growth factor receptor 2 (HER2) enriched cancers. This insight has been developed into a clinical framework that classifies breast cancer into five distinct types on a patient-to-patient basis, depending on the enrichment of HER2 and the expression of hormone receptors, as well as the triple-negative phenotype that shows no overexpression of HR receptors, which include estrogen and progesterone receptors, and lacks HER2 overexpression [7]. A multitude of genotypic, transcriptomic, and protein level alterations are responsible for the heterogeneous phenotypes seen in breast cancer. Identifying these phenotypes and elucidating their underlying mechanisms is essential not only for directing patient care but also for the development of effective targeted therapies (Table 1).

## 4. Genetic Driver Mutations

The advent of sequencing technologies has allowed the stratification of histologically similar cancer cases into subtypes based on common genetic driver mutations. This understanding of mutations in oncogenes and tumor-suppressor genes allows a patient’s disease to be more accurately prognosticated, predicting their cancer’s resistance to commonly employed therapies.

Continuing with the example of breast cancer, even in the inaugural genetic events in tumor formation arises heterogeneously. Low-grade breast lesions are often characterized by genetic alterations that increase the expression of the HR phenotype. This can occur through 6q25 gene amplification, which increases the copy number of the *ESR1* gene that encodes estrogen receptor alpha (ERα) [8]. It has also been demonstrated that certain *ESR1* mutations cause greater protein stability, giving rise to greater ER abundance in these tumors [9]. While the specific mechanisms that govern HR expression differ, the mutation-induced overexpression of ER accelerates the progression of breast cancer cells through the binding of estrogen. Estrogen signaling then induces intracellular transcription factors associated with cancer growth and proliferation [10]. Drugs that inhibit estrogen production or block the interaction of estrogen with ER, such as aromatase inhibitors or tamoxifen, respectively, have proven highly effective in many breast cancer patients [11,12]. However, point mutations in *ESR1* can induce a dimerized phenotype of ER that allows for constitutive activation without estradiol binding [13]. These mutations would allow hormone-independent proliferation, conferring resistance to anti-estrogen therapies [14].

Concerning HER2, its gene *ERBB2* has been found to be genetically amplified in approximately 15% to 20% of breast cancer patients [15,16]. HER2 can stimulate cancer cell growth through the PI3K–AKT–mTOR pathway [17]. However, HER2 has no known activating ligand but instead heterodimerizes with other ligand-binding HER family members, allosterically activating the HER2 receptor tyrosine kinase (RTK) [18]. Along with triple-negative breast cancer, HER2-positive disease was long considered to be among the most aggressive breast cancers, having limited treatment options. The introduction of the anti-HER2 monoclonal antibody, trastuzumab, has shown marked survival benefits for patients with HER2 upregulation and demonstrated suppression of disease recurrence [19]. Trastuzumab inhibits the extracellular domain of HER2 and suppresses intracellular signaling of HER2 target genes [20]. However, a number of genetic mutations that modulate either the expression of HER2 or cause constitutively active versions of the RTK can emerge due to therapy-induced acquired resistance [21].

This genetic heterogeneity in breast cancer patients is why molecular testing is now routinely employed to aid the characterization and treatment of the disease. Both progesterone receptor and estrogen receptor overexpression are assessed via immunohistochemistry (IHC) in order to inform the decision to use endocrine therapy for breast cancer. In situ hybridization can be used to determine the effectiveness of trastuzumab or other anti-HER2 therapies by assessing the amplification status of *ERBB2* in the genome [22]. Additionally, sequencing is used to determine the presence of *BRCA1* and *BRCA2* germline mutations, which predict the response to PARP inhibition [23].

Next-generation sequencing (NGS) technology will be of particular importance to comprehending the unique genetic features of a patient’s cancer, allowing clinicians to differentiate between patients with subtle yet meaningful molecular features. The high nucleotide resolution of high sequencing depth coverage NGS allows for detecting covert molecular events that can guide crucial treatment decisions [24]. A study testing massively parallel DNA sequencing of paraffin-embedded clinical specimens from over 2000 patients demonstrated that NGS provided actionable therapeutic intelligence to 76% percent of patients, constituting a three-fold improvement over what could be achieved by conventional diagnostic testing of these tissues [25]. There are enormous financial and practical challenges in broadly providing NGS services to cancer patients. However, a further study demonstrated that even cancer-agnostic approaches in unselected patients are feasible [26]. Although currently beyond the capabilities of many institutions, the rapidly decreasing per-base cost of whole-exome sequencing and astute enrichment strategies may greatly increase the number of non-trial patients who could benefit from NGS diagnostics. Additionally, NGS data from patients greatly aids the discovery of novel biomarkers and potential therapeutic targets, further advancing the development of precision oncology and ultimately leading to more effective and personalized cancer care [27].

**Table 1:** Technologies for assaying interpatient genetic heterogeneity.

Technology	Principle	Application	Ref.
IHC	Chromogenically visualized antibody	Tissue level visualization of protein expression, including mutant proteins	[4]
In Situ Hybridization	Complementary DNA/RNA probes	Detection of genetic variants and copy number alterations at single-cell resolution	[22]
PCR	DNA amplification using primers	Highly sensitive detection of wide range of genetic variants including repeat expansions	[28]
Gene Panels	Hybridization probes arranged in array	Detection of select arrangement of genes with deep coverage	[29]
NGS	Amplification and massively parallel sequencing of sample DNA	Large scale sequencing for comprehensive genomic analysis and detection of novel genetic signatures	[5,24,25,26,27]

## 5. Epigenetic Heterogeneity

NGS technology alone, however, will not provide enough data to guide future precision therapy development since a significant degree of clinical heterogeneity cannot solely be explained by mutational differences. Interpatient heterogeneity is not merely limited to diverse genotypic presentations. A host of non-mutational mechanisms are also responsible for the disparate manifestations of cancer. Epigenetic heterogeneity, for instance, describes the variability in epigenetic modifications, which include changes in DNA methylation, histone modification, and non-coding RNA expression. Although these modifications do not alter the DNA sequence, they can significantly impact gene expression, thereby driving phenotypic changes. A variety of endogenous physiological processes, such as embryonic development or long-term memory formation, rely on epigenetic regulation [30,31]. A considerable body of work demonstrates that epigenetic regulation is a biological mechanism that cancer harnesses to provide a further degree of heterogeneity and, thereby, greater evolutionary latitude. In fact, non-mutational epigenetic reprogramming has been introduced as a hallmark of cancer that provides a mutation-less plane of cancer evolution and therapeutic variability that cannot be captured by genetic sequencing techniques alone [32].

A convincing example of epigenetically driven heterogeneity in cancer is the phenomenon of epithelial-to-mesenchymal transition (EMT). EMT provides a reversible and dynamic mechanism for cancer cells to transition to states that display mesenchymal characteristics and have greater potential for invasiveness [33]. EMT is an important feature of many cancers that promotes tumor plasticity, drug resistance, and aggressiveness, causing two otherwise similar cancers to behave differently over time. EMT is characterized by a loss of the epithelial adhesion protein E-cadherin and an increase of the mesenchymal N-cadherin. E-cadherin has been shown to be epigenetically regulated by the major EMT regulation factor, *ZEB1*. In addition to acting as a DNA binding transcription factor for EMT, *ZEB1* also represses E-cadherin by recruiting the chromatin-remodeling protein BRG1 [34]. *ZEB1* has also been shown to induce a positive feedback loop on itself through a histone methyltransferase, *SETD1B* [35]. Additionally, *SNA1L*, another central regulator of EMT, has a well-established role in epigenetically silencing E-cadherin [36]. EMT can also confer drug resistance [37]. The EMT transcription factors, *ZEB1*, *SNA1L*, and *SLUG* can induce resistance to platinum-based chemotherapy in breast, ovarian, colon, and pancreatic cancer, providing a selection advantage to this epigenetic state [38,39]. In breast cancer, EMT can lead to methylation of *ESR1* via a SNAI2–DNMT3B complex, decreasing ER expression and promoting a phenotype with an unfavorable prognosis [40,41].

Another highly clinically relevant example of epigenetic heterogeneity is the hypermethylation of the *MGMT* gene in glioblastoma. The *MGMT* gene encodes for O6-methylguanine-DNA methyltransferase, which protects the genome by removing alkyl group adducts from the O^6^ of guanine [42]. A methyl group on the O^6^ of guanine results in faulty pairing to thymine during DNA replication. In an invariably ill-fated attempt to resolve this discrepancy, the mismatch repair pathway fails to remove the methylated guanine, eventually leading to a double-stranded DNA break, which can cause cell death via apoptosis. The alkylating agent Temozolomide causes DNA damage by adding methyl groups, among other sites, to the O^6^ of guanine. However, the corrective role of *MGMT* means that temozolomide is generally only effective in *MGMT*-deficient cancers [43]. *MGMT* is heavily regulated through methylation of the CpG islands in the gene promoter, inhibiting transcription of *MGMT* [44]. *MGMT* methylation is observed in 34–45% of glioblastoma cases [45,46]. However, it must be noted that while promoter methylation is the primary mechanism of *MGMT* regulation, gene body methylation, histone modifications, and transcriptional activators and repressors also play an important role [47]. There is a documented discrepancy between *MGMT* status and gene promoter methylation, whereby *MGMT*-deficient cells may not show promoter methylation, emphasizing the need for diagnostic evaluation of *MGMT* status at the transcriptomic or protein level. Nonetheless, epigenetic mechanisms are incredibly important in producing heterogeneous clinical phenotypes in glioblastoma.

It follows that epigenetically regulated drug-resistant phenotypes can confer an evolutionary advantage that selects for a specific epigenetic tumor cell state. These epigenetic changes may only be transient in nature and cannot be evidenced by genomic alterations, making them particularly hard to characterize. Heterogeneity of the epigenome may explain why patients see disparate responses to therapies that a purely genetic model cannot explain. These examples demonstrate that clinically relevant cancer subtypes are regulated by the epigenome, underlining the importance of multimodal diagnostic techniques that can augment DNA sequencing data to evaluate cancer tissue phenotypes (Table 2).

Emerging technologies can profile genome-wide DNA methylation, histone modification, and chromatin accessibility, offering a promising means of deciphering the heterogeneity of the cancer epigenome directly and at scale [48,49,50,51]. Through treating genomic DNA with sodium bisulfite, unmethylated cytosines are converted into uracil, creating a chemical distinction between methylated and unmethylated DNA. Methylation sensitive PCR has been demonstrated as a viable means of investigating the methylation status of tumor DNA CpG islands [52]. Sequencing of sodium bisulfite treated DNA has also been demonstrated at the whole genome level, uncovering the status of super-enhancer DNA methylation throughout the genome [48]. Chromatin immunoprecipitation (ChIP) is another assay that can assess epigenetic modifications in cancer by quantifying histone modifications throughout the genome. ChIP uses antibodies to pull-down DNA with a particular histone modification. This DNA can then be sequenced in bulk to a genome-wide mapping of histone modification (ChIP-Seq), or the technique can be combined with PCR to investigate a particular site (ChIP-qPCR) [53,54]. Recently, single-cell ChIP-seq has been validated as a viable strategy for uncovering heterogenous chromatin states within breast tumors [55]. Finally, ATAC-seq is an assay that unveils chromatin accessible DNA through the use of an enzymatic treatment [56]. A genome-wide assessment of chromatin accessibility performed on 410 Cancer Genome Atlas specimens has been integrated into the TCGA database, allowing extensive exploration of an entirely new dimension of the noncoding genome [51].

**Table 2:** Technologies for assaying interpatient epigenetic heterogeneity.

Technology	Principle	Application	Ref.
Methylation sensitive PCR	Amplification of sodium bisulfite treated DNA using primers	Highly sensitive and specific determination of the methylation status of CpG in particular genomic regions	[52]
Bisulfite Sequencing	Amplification and massively parallel sequencing of sodium bisulfite treated DNA	Genome-wide characterization of gene regulatory status through methylation assessment of promoter regions	[48]
ChIP-seq/PCR	Immunoprecipitation of histone modified DNA	Assessment of the genome-wide (seq) or single region (PCR) histone modification profile	[53,54,55]
ATAC-seq	Assessment of transposase- accessible genome regions	Genome-wide investigation of chromatin accessibility	[51,56]

## 6. Transcriptional Regulation

In addition to the epigenome, transcriptional regulation through DNA-binding transcription factors provides a further dimension of heterogeneity that contributes to the variety of cancer phenotypes. The inductive dynamics of these transcription factors, similar to genetic selection, can act as a medium for Darwinian evolution, leading to the emergence of a prevalent phenotype with no sign of genetic alteration.

The phenomenon of neuroendocrine differentiation (NED) is an example of the inductive interchanges of transcription factors catalyzing phenotypic plasticity. NED is a histological transformation that may emerge in the later stages of disease progression that is often documented in advanced prostate and lung cancer [57]. In prostate cancer, a blockade against androgen receptors (ARs) with androgen pathway inhibitors (APIs) can interfere with the main transcriptional actor of the disease. In the metastatic setting, however, these therapies will ultimately render the disease API-resistant. NED is a mechanism with which prostate cancer cells evade the inhibitory effects of API therapy, turning to AR-independent drivers of proliferation.

Similarly, targeted therapies such as EGFR inhibitors can induce NED directly in lung cancer. However, NED has also been documented to arise spontaneously due to selective pressure and the growth advantage provided by the neuroendocrine (NE) phenotype [58]. NED is associated with poor survival outcomes due to the aggressiveness of the disease and lack of adequate treatment options.

The exact causes of NED are multifaceted and remain elusive, but transcriptional reprogramming plays a significant role. In vitro experiments suggest that AR exhibits unique transcriptional activity in cells that have developed resistance to APIs, with one study revealing over 2000 unique AR-DNA binding sites in cells with acquired resistance to the API enzalutamide [59]. AR was also found to bind closer to stem cell and neuronal transcription factor motifs in resistant cells, correlating with an enrichment of RNA from their corresponding transcriptional pathways. In redifferentiation, NE prostate cancer also eventually loses AR expression, entirely relying on alternate pathways for proliferation [60]. The activation of a myriad of transcriptional drivers has been linked to NED, including N-Myc, SOX2, BRN2, ASCL1, OCT4, and more [61].

It is also critical to emphasize that NED is not solely reliant on transcriptional regulation. An interconnectedness of transcriptional, epigenetic, and genetic changes is essential to the NED process. For instance, the histone trimethylation enzyme EZH2 is also commonly overexpressed in NEPC, underscoring the close interaction between transcriptional and epigenetic alterations in cells undergoing NED [62]. Further studies have even pointed to specific miRNAs as drivers of NED [63]. Genetic events also appear to assist the transition to NEPC. While the phenotypic transformation of NED itself is driven by aberrant transcription, studies investigating the prevalence of genomic alterations in NEPC frequently report *TP53* and *RB1* mutation or loss [64]. A possible explanation is that the wild-type function of these proteins interferes with the lineage transformation process in terminally differentiated cells, which is a hypothesis supported by research describing p53 and Rb-related suppression of NED in mouse models [65]. However, genetic alterations are not ubiquitous, as illustrated by a minority of metastatic castration-resistant prostate cancer presenting as neuroendocrine while retaining diffuse Rb expression tested via IHC [66].

Analyses of patient samples have uncovered several molecular subpopulations present in NE cancer. One study analyzing metastatic castration-resistant prostate cancer (CRPC) patient samples found fairly consistent chromatin states across NE tumors but identified a bifurcation between ASCL1 and NEUROD1-expressing cells [67]. These NE subtypes could exist in the same tumor concurrently, representing intratumoral and interpatient heterogeneity. A similar study corroborated these results, noting two groups between and within patients: one with high ASCL1 expression and one with functional secretory NE markers like chromogranin A [68]. The authors also suggest that cells may transition from the ASCL1 subtype to the secretory subtype.

One effort to profile oncogenic kinase activation in NEPC discovered a high degree of heterogeneity in kinase activation between patients but a homogeneity at the intrapatient level [69]. The lack of heterogeneity between metastases is promising for the efficacy of targeted kinase inhibitors, as multiple lesions may respond to the same therapy. Still, these drugs have yet to demonstrate sweeping success in the clinic. For instance, a phase two clinical trial for an Aurora kinase inhibitor found limited success overall but reported some exceptional responders with high expression of relevant biomarkers [70]. Preclinical work has also identified RET kinase as a potential target in NEPC [71]. Further research towards discerning the relevant drivers of NED will inform the selection of optimal treatment for a specific patient and facilitate the discovery of novel targets that will allow for new ways to interfere with or reverse NED. Because of the interpatient heterogeneity present in tumors having undergone NED, personalized treatment based on molecular tumor signatures will be paramount in providing patients with optimal treatment.

## 7. Tumor Microenvironment Heterogeneity

Beyond cancer cells themselves, the tumor microenvironment (TME) has been established as a major contributor to solid tumor growth and metastasis, dramatically affecting treatment outcomes. The TME includes stromal cells such as fibroblasts, mesenchymal stem cells, endothelial cells, and immune cells [72]. Cancer cells play an active role in modulating the TME to support their survival, and elements of the TME have served as the basis for novel therapeutics such as angiogenesis inhibitors and immune checkpoint blockade therapy [73].

One of the most well-established delineations between the TMEs of different patients is “hot” versus “cold” immune microenvironments. Hot tumors exhibit a high degree of immune infiltration, while cold tumors are relatively devoid of immune activity. Several factors influence a tumor’s specific immune microenvironment, including the profile of signaling molecules present in the TME [74]. Studies investigating the cytokine landscape of tumors have identified correlations between cytokine expression and disease progression for molecules like IL-6 and IL-1 receptor agonist [75,76]. A study of heterogeneity in glioblastoma revealed that the distinct RNA profiles of tumors led to differential immune infiltration, with more mesenchymal tumors exhibiting a greater immune presence [77]. The abundance of specific immune cell cohorts has been shown to drastically affect overall prognosis and immunotherapy response in patients [78]. A hot TME is often associated with pro-inflammatory indications and the presence of tumor-infiltrating and CD8-positive T lymphocytes, while a cohort of suppressor and regulatory T cells impeded an anti-tumor immune response. Studies in clear cell renal cell carcinoma have shown potent immune responses driven by tumor-immune cell infiltration, even without high degrees of tumor mutation burden [79]. Eliciting a tumor-immune response is a highly context-dependent process that clearly relies on many non-genetic factors.

Stromal elements of the TME also contribute to interpatient heterogeneity. For example, an analysis of cancer-associated fibroblast (CAF) subtypes in breast cancer found differential CAF marker expression depending on the specific molecular subtype of breast cancer [80].

Cancer stem cells, a subset of highly self-renewing cells within a tumor, are also a significant contributor to heterogeneity in the TME. Cancer stem cells have the potential to maintain and regenerate a tumor by harnessing their replicative potential to spawn new cancer cells [81,82]. One study in lung tumors found differential expression of stemness markers Nanog and CD133 across samples, suggesting interpatient heterogeneity of cancer stem cells [83]. The scarcity of stem cells in tumors and their potentially fleeting phenotypes makes them notoriously difficult to study. Nonetheless, their contribution to tumor heterogeneity is understood to arise from their ability to provide a reservoir of cellular replication from which tumors can adapt to outside factors and recur [84,85].

Another feature characteristic of the TME is hypoxia with a reduced pH. The degree of hypoxia varies by tumor type and organ, typically correlating with a worse prognosis [86]. Hypoxic conditions have several effects on the TME, including differential gene expression in cancer cells and the formation of new blood vessels through angiogenesis. One study of liver tumors and their metastases found that lesions with a hypoxic TME presented with poorer patient survival. These tumors increased expression of *SLC2A1*, the gene encoding the GLUT1 transporter [87].

In summary, the complexity of various components within the tumor microenvironment provided by immune resistance mechanisms, stromal cells, cancer stem cells, and hypoxia creates powerful selection pressure that can heterogeneously drive cancer evolution and alter the response to therapeutics. Building a stronger understanding of these effects and elucidating the reasons behind differential therapeutic responses will help in the development of more effective drugs and more robust treatment regimens.

## 8. Intra-Tumoral Heterogeneity

Advancements in molecular analytical techniques have significantly deepened our understanding of cancer’s genetic, epigenetic, and transcriptional heterogeneity (Table 3). This comprehensive cataloging of cancer’s complex yet recurrent patterns of heterogeneity holds the promise of enabling more precise and effective targeted therapies. Clinicians can harness these molecular insights to refine diagnoses and optimize treatment strategies tailored to each patient’s unique cancer profile. However, heterogeneity is not only observed across different patients but also within individual tumors. The various mechanisms of heterogeneity previously described between patients are also found intratumorally within single lesions. The genetic diversity of single lesions has long been known, with karyotyping studies revealing the disparate chromosomal aberrations observed within tumors [88]. Single-cell RNA sequencing technology and spatial transcriptomics have shed light on the transcriptomic heterogeneity observed within individual lesions. This phenotypic diversity is an inherent property of solid tumors and poses a significant challenge to achieving durable and curative treatment responses [89].

Studies on the random X-chromosome inactivation that occurs in female human blastocysts proved that most non-virally induced cancers are monoclonal in origin [90,91,92]. Pioneering studies in colorectal cancer by Vogelstein and colleagues outlined the distinct mutational path a cell must take to eventually develop into a metastatic malignancy [93]. These genetic steps are rare; therefore, the stepwise progression takes years. As mutations accumulate, these cells acquire a near-unlimited replicative potential and genomic instability, substantially increasing the likelihood and rate of further mutations. Aided by a high proliferative rate, a growing lesion serves as a genetic reservoir that allows sub-clonal cells containing heterogeneous driver genes to emerge in time.

Models of cancer evolution show that the development of heterogeneity occurs through new mutations, which occasionally confer a growth advantage, resulting in a clonal sweep of a more fit clone. Importantly, heterogeneity is not a static state but is obscured by spatial and temporal competition between clones [94]. The dynamic nature of cancer cell populations complicates accurately capturing the diversity within a lesion as, not only the technical sophistication to detect these clonal changes is necessary, but also the means to quantify and interpret this dynamic data set. Concepts from population genetics can be used to quantitatively describe the degree of genetic heterogeneity within a population of cells, thereby describing the evolutionary course of a cancer. For example, a study analyzing esophageal biopsy specimens from patients with Barrett’s esophagus used the Shannon index, a population genetics metric for quantifying the diversity of a population, to predict the progression of Barrett’s esophagus to esophageal cancer [95]. In the study, increased Shannon indexes, describing a higher genetic diversity, correlated with a higher propensity for malignant progression. However, in this case, with a measure as broad as the Shannon index, it is difficult to associate the increased genetic diversity with any discreet mechanistic and causative phenomena. Therefore, statistical variables, without the identification of individual molecular adaptations, can only offer actionable data to a limited degree.

Using more targeted and high-fidelity analyses of cancer heterogeneity can enable investigators to recreate the evolutionary path that a cancer took. Phylogenetic reconstruction then allows the identification of cancer addictions and can inform targeted therapy selection. Non-small cell lung cancer (NSCLC) is an example of a malignancy that displays widespread intra-tumoral heterogeneity. An analysis of the mutational profile from multiple sites of resected NSCLC primary and metastatic lesions revealed widespread heterogeneity of non-silent mutations within lesions [96]. Phylogenetic reconstruction identified that mutations in *BRAF*, *EGFR*, *RB1*, or *TP53* were consistently mutated in primary and metastatic samples, coinciding with their role in early NSCLC development. However, mutations in *PIK3CA*, for instance, stemmed from sub-clonal populations found only in parts of individual lesions. The authors point out that, hypothetically, a single-site biopsy that coincidentally probed the *PIK3CA* mutant region of the tumor could suggest the use of a PI3K/mTOR pathway inhibitor. Alarmingly, the heterogeneity of *PIK3CA* status in this particular lesion would render such a therapy ineffective because of the abundance of resistant subclones. In this case, there is a chance that a single-site biopsy would have failed to provide an accurate molecular picture of the cancer, potentially leading to an ineffective clinical decision.

Studies in head and neck squamous cell carcinoma (HNSCC) have demonstrated that non-mutational epigenetic levels of heterogeneity are also present intratumorally [97]. Single-cell RNA sequencing from patients with HNSCC demonstrated a subpopulation of cells in a partial epithelial-to-mesenchymal (p-EMT) state that was genetically indistinguishable from normal HNSCC cells. This population was defined as p-EMT because of the presence of several classical EMT markers while lacking substantial upregulation of the transcription factors *ZEB1*, *SNAIL,* and *TWIST*. These p-EMT cells lined the invasive and growing borders on the outside of the tumor. An in-vitro model using SCC9 HNSCC cells demonstrated p-EMT positive subpopulation that showed higher invasiveness than non-p-EMT SSC9 cells. Interestingly, when SCC9 cells were FACS sorted for p-EMT positive and negative populations, both groups reverted to an unsorted distribution within four days in culture. The maintenance and even regeneration of this tumoral heterogeneity in vitro, in the absence of stromal paracrine factors, indicates a cancer-cell intrinsic mechanism of intratumoral heterogeneity.

Attempts to model disease in vitro frequently mimic the heterogeneous cellular populations observed in the clinic. Returning to the example of neuroendocrine differentiation, many of these models involve genetic and transcriptomic modifications to mimic the protein expression levels observed in patients. One such model involves the delivery of the dominant negative p53, myrAkt1, *RB1*-shRNA, c-Myc, and Bcl-2 to basal cells taken from patients with castration-resistant prostate cancer [98]. When organoids derived from this model were grown in vitro and implanted into mice, they eventually expressed the NE markers synaptophysin and NCAM1. Demonstrating the inherent heterogeneity in the NED process, these organoid experiments produced two distinct lineages over time, one which expressed the NE transcription factor ASCL1 and the other which expressed the typically non-NE transcription factor ASCL2. Another approach to model NED is to use mice to mimic its clinical development in vivo. Xenografts grown in castrated mice and xenografts treated with enzalutamide have both generated cell lines that recapitulate the molecular heterogeneity of NEPC [99,100].

An analysis of intra-tumoral heterogeneity in renal carcinoma using multi-site biopsies revealed that although truncal driver mutations such as in *VHL* are conserved, over 60% of mutations were not detected across all regions of the primary tumor [101]. Their study revealed clonal populations containing different inactivating mutations in *SETD2*, *PTEN*, and *mTOR* from spatially separated sites, demonstrating not only genetic heterogeneity but also an instance of convergent evolution in renal carcinoma. This study again shows another example where single-site biopsies, common in clinical practice, may lead to a drastic underestimation of intra-tumoral heterogeneity, causing a failure of targeted agents.

This study also demonstrated that it was one single site of the primary renal carcinoma that was the progenitor to all of the metastatic lesions. Since a single clone of the primary lesions was responsible for all metastases, there is evidently an interdependence between intra- and inter-tumoral heterogeneity. In addition to highlighting the value of analyzing multiple biopsy sites, this study also suggests that single-cell transcriptomics may be a powerful tool for studying intratumoral heterogeneity by offering detailed insights into the genetic and transcriptional dynamics within individual lesions. This technology is tremendously expensive and therefore not likely to be widely adopted for clinical use in the near future. However, single-cell transcriptomics allows for the molecular analysis of multiple subclones within one single tumor and is an invaluable tool for understanding heterogeneity-driven drug resistance and for therapeutics discovery [102].

## 9. Spatial Omics

Perhaps the most pertinent advance in the study of intratumoral heterogeneity is the rapid development of spatial multi-omics. Similarly to single-cell analyses, spatial techniques can be used to characterize the heterogeneity within a specimen, with the added benefit of mapping molecular characteristics to specific spatial regions within a tissue sample. The power of spatial techniques in deciphering tumor heterogeneity lies in their ability to visualize heterogeneous clonal cell populations and uncover communication networks between different cell types of both malignant and non-cancerous origin. Currently available spatial techniques rely on antibodies or nucleic acid primers to target molecules of interest and can be employed to analyze the proteome, transcriptome, and even the epigenome [103].

Both in the clinic and experimentally, antibody-based labeling of protein in tissues samples is the most established technique of spatial proteomic analysis. Immunohistochemistry (IHC), for instance, relies on an antibody to bind target proteins, allowing for the subsequent identification of positive cells under a light microscope. For not only higher sensitivity but also the visualization of a broader range of proteins, immunofluorescence (IF) may be employed. IF is a technique in which antibodies care conjugated to a fluorescent signal rather than a chromogenic signal, allowing for the analysis of multiple channels simultaneously [104]. Both techniques have proven invaluable in identifying the distribution of protein expression and cell types over tumor specimens.

Spatial transcriptomics discerns the RNA expression levels of target genes or, if sequencing is employed, the entire transcriptome. Several varieties of spatial transcriptomics are available today, including in situ imaging, spatial indexing techniques, or microdissection processes [105]. With in situ techniques, such as RNA in situ hybridization (RNA-ISH), a slide is labeled with primers that hybridize with a specific ligand, emitting a unique signal from a specific region of the sample. This technique allows for the visualization of differential RNA expression across the sample, analogous to the way immunohistochemistry identifies regions of protein expression. Methods like RNA-ISH are able to detect subcellular localization of transcripts but are limited by the number of primers that can be employed at a time. Alternatively, spatial indexing techniques implement sequencing to analyze thousands of genes in a given sample. Technologies such as 10X Genomics’s Visium system, use chips pre-coated with microscopic matrix of “spots” containing locally barcoded RNA primers [106]. A fixed tissue sample can be applied to these chips, hybridizing the sample transcriptome with barcoded primers that are indexed to specific coordinates on the slide. Following cDNA synthesis, sequencing reveals both the transcript and its location on the sample via the barcode. This allows the generation of spatial heat maps of tissue gene expressions at a resolution of 1–10 cells.

Further methodological advances allow for the identification of even more nuanced forms of intratumoral heterogeneity, such as the distribution of epigenetic signatures across a sample. Epigenomic MERFISH builds on the ISH-based technology MERFISH, which uses combinatorial fluorescent signals to survey a larger number of genes than is possible with traditional single-molecule ISH [107]. Epigenomic MERFISH allows for the spatial visualization of epigenetic marks across a tissue sample and the characterization of promoter activity. Instead of a primer, epigenetic MERFISH uses an antibody to selectively target histone marks, and adopts a similar method to CUT&Tag to tag DNA with T7 promoter and sequencing primers, followed by in situ transcription and MERFISH imaging [108].

Each spatial technique has its advantages and disadvantages with respect to sensitivity, throughput, resolution, and cost. Proteomic methods, for instance, can offer great spatial resolution but may be limited by poor antibody-target sensitivity and specificity. Additionally, even with careful fluorophore selection, proteomic panels are limited by the number of proteins that can be investigated simultaneously. While sequencing-based transcriptomic methods allow the capturing of the entire transcriptome, these methods come at a significantly higher cost and require sophisticated data analysis. Additionally, the goal of reliably achieving single-cell resolution through spatial RNA transcriptomic techniques has remained elusive. Attempts to address this issue through computational deconvolution methods are currently under investigation. For instance, statistical or deep learning models may improve the resolution of and remove noise from spatial imaging techniques [109,110,111]. Iterative advances in both the physical technologies and computational deconvolution algorithms will help improve the accessibility and utility of these analytical techniques, ultimately potentiating their investigative power.

## 10. Multi-Omics

While many of these techniques are immensely powerful in and of themselves, the ability to combine spatial data types through multiplexing greatly enhances their potential. Spatial transcriptomic systems similar to Visium can be combined with immunofluorescence to visualize RNA transcripts and the expression of a limited set of proteins [112]. Another set of techniques relies on physically isolating regions of interest (ROIs) on the sample prior to sequencing. Nanostring’s GeoMX, for instance, combines fluorescent antibodies with RNA-ISH probes with transcript-specific barcodes. The user then selects regions of interest via immunofluorescence microscopy to direct the UV light-mediated selective cleavage of barcodes at the region of interest. Subsequent quantification allows transcript levels to be traced to their ROI in the original sample, in addition to the protein-level data that the antibodies provide [113]. Because antibodies can also be used to target specific epigenetic marks on histones, spatial epigenetic methods can be combined with RNA sequencing to correlate histone marks around particular genes with their corresponding mRNA expression in local regions of a sample. This technique allows for the visualization of how epigenetic marks affect transcription in space, shedding light on how epigenetic diversity across a tumor may alter its characteristics.

Spatial multi-omics are transforming the way tumors are studied and offer the potential to deepen our understanding of tumor evolution and cellular interactions. The previous example of spatially deciphering epigenetic signatures and RNA transcripts can reveal how epigenetic modifications affect local RNA expression. This method provides two spatial-omic levels which can validate one another and determine exactly how one is correlated with the other. This level of detail is particularly relevant in tumor tissues, where the effects of severely altered epigenetic profiles are still being revealed [114]. Additionally, the ability of spatial transcriptomic techniques to characterize immune cell populations could provide insight into the complex interactions between tumor and immune cells. Spatial multi-omics can help uncover the characteristics of the TME that are conducive to antitumor immune activity and those which suppress the immune response. Further, initiatives such as the NCI-funded Human Tumor Atlas (HTAN) seek to collect and integrate spatial multi-omics data on a multitude of tumor types to paint a spatial and temporal pictures of tumor development, with the public distribution of this data helping to facilitate new discoveries [115].

Although, for the moment, the clinical application of spatial techniques is mainly limited to IHC, the insights provided by spatial multi-omics may provide physicians with invaluable prognostic and diagnostic information. The complexity and scale of data produced by these technologies would make the widespread clinical rollout of spatial multi-omics a formidable challenge. Integration of machine learning models, trained on vast sets of spatial molecular data and correlated with patient treatment outcomes, with patient biopsies to inform optimal treatment decisions would ultimately provide a highly comprehensive and personalized approach to cancer care [116,117].

**Table 3:** Technologies for assaying intratumoral heterogeneity.

Technology	Principle	Application	Ref.
Multi-site biopsies	Genomic or proteomic analysis of spatially distinct tumor samples	Uncovering heterogenous clonal populations spread out within a patient’s tumor	[101]
Single-cell RNA-seq	Captures gene expression at the single-cell level	Analyzing cellular diversity, identifying cell subtypes, and studying differential gene expression across cell types	[97,102]
Immuno- fluorescence	Antibodies conjugated to fluorescent dyes to detect protein expression	Localizing specific proteins within cells or tissues and studying protein–protein interactions	[104]
Spatial transcriptomics (e.g., Visium)	Barcoded RNA probes to map RNA-seq data to spatial locations in tissue	Visualizing gene expression in tissue sections, linking gene activity to tissue histology	[106]
Spatial epigenomics (e.g., epigenetic MERFISH)	Antibody-based probing of histone modifications with concomitant in situ transcriptions	Highly multiplexed spatial mapping of epigenetic markers and chromatin organization at high resolution in tissues	[107,108]
Spatial multi-omics (e.g., GeoMX)	Combines IF with spatial transcriptomics through UV cleavage of ROIs	High-throughput spatial profiling of both transcriptome and protein panel in a tissue sample	[113]

## 11. Inter-Tumoral Heterogeneity

Since pre-existing subclones of the primary lesion are usually responsible for forming metastatic lesions, there is evidently an intimate connection between intra- and inter-tumoral heterogeneity [93]. Therefore, heterogeneity also presents between the individual lesions of a single patient. The importance of understanding the intertumoral heterogeneity between metastatic lesions is underscored by the clinical reality that metastases to the liver, brain, lung, or bone are the cause of cancer deaths for the majority of patients [1]. Since heterogeneity is a common driver of resistance and because many patients die due to recurrent metastatic lesions, gaining an understanding of the heterogeneity between and within metastatic lesions is of greatest clinical interest (Table 4).

Given that the metastatic lesions arise from one single sub-clonal population of the primary lesion, it is conceivable that there is initially low inter-metastatic heterogeneity [118,119]. In a study of patients with at least two treatment-naive metastases, it was found, using exome-wide sequencing, that all functional driver mutations were consistent across metastatic lesions [94]. This consistency among metastatic lesions is echoed by clinical correlates that show that it is common for all metastatic lesions to show similar initial responses to targeted therapies. In a cohort of 33 melanoma patients, each having at least two lesions as well as confirmed *BRAF* V600E mutations in their primary tumors, of the 27 patients that responded to targeted therapy (dabrafenib, trametinib, GSK2141795), 23 showed a consistent response across all lesions. Although there were differences in magnitude and timing of response, likely dependent on tumor microenvironment factors such as vascularity, these studies confirm that a targetable driver mutation in one metastatic lesion is usually shared by all other lesions in a patient.

Even patients who see a response across all metastatic lesions often relapse on targeted therapies due to resistance mechanisms. Similarly to intra-primary heterogeneity, therapy resistance occurs due to the selection of pre-existing sub-clonal populations within a metastatic lesion [120,121]. Studies in *EGFR* mutant lung cancer show that sub-populations with *MET* amplification-induced activation of PI3K/AKT signaling possess resistance to EGFR kinase inhibitors prior to therapy [122]. Evidence shows that by the time metastatic lesions have reached the size to be visible on radiological imaging, they are large enough to already contain sub-clonal populations with resistance to many drugs [121,123]. Theoretically, by employing novel combination therapies that attack multiple pathways simultaneously, there is a chance to completely eradicate these lesions, leading to a curative response.

However, this outcome may be complicated by several non-genetic features of heterogeneity that impede a consistent response, even to combination therapies. The lack of genetic evidence for metastases offers a clue as to the effect of non-genetic variability across metastatic lesions. Although discrete mutations that are associated with cancer formation and progression have been countlessly described, a clear genetic basis for metastasis has remained elusive. The fact that no such connection can be proven, even with the rapid emergence of high-throughput sequencing technology, suggests that the metastatic cascade may be reliant on non-genetic factors. One of the prevailing theories of cancer cell metastasis is that tumor cells use a nuanced process whereby tumor evolution selects for cells that possess an epigenetic or transcriptional potential for greater adaptability to novel environments [1]. This increased survivability may also confer a heterogeneous response to treatment once these metastatic lesions have formed. The ability to react to contextual signals that enables metastasis may also contribute to heterogeneity-driven drug resistance.

Once these resistance mechanisms in metastases develop, it is hard to predict their evolutionary path. Biopsy of multiple metastatic lesions to observe the evolutionary change may also be clinically infeasible. The sequencing of cell-free DNA (cfDNA) allows for the analysis of DNA from blood plasma [124]. This procedure could be performed repeatedly with minimal risk to the patient, allowing for continual monitoring of a patient’s course, especially throughout treatment regimens. Sequencing of cfDNA can allow for the diagnosis, prognosis, and selection of treatment regimens for patients with heterogenous disease by sampling cfDNA systemically [125]. However, the relative scarcity of tumor DNA in circulation can hamper the acquisition of rare mutations, especially considering that only a small fraction of sampled cfDNA is from cancer cells and not from healthy tissue. Therefore, clinicians must be acutely aware of the limitations of cfDNA sequencing and its ability to portray a complete and accurate picture of a patient’s disease.

Nonetheless, the 2016 approval of liquid biopsy for *EGFR* cfDNA has since proven its clinical utility, providing actionable intelligence for NSCLC in a long-term study [126]. Furthermore, a recent study demonstrated that the accuracy and utility of liquid biopsies can still be greatly improved upon by introducing computational error correction techniques that allowed for 92% sensitivity and 96% specificity for detecting *EGFR* mutations in patients with NSCLC, which is a considerable improvement over previous liquid biopsy performance [127]. The fact that a liquid biopsy can measure DNA that sheds into the bloodstream from any cancerous lesions within a patient means that a more holistic mutational picture of the patient’s course can be formed than can be obtained from a single biopsy. Although currently only FDA approved for identifying EGFR mutations in NSCLC, liquid biopsies promise to provide a non-invasive means of analyzing inter- and intra-tumoral heterogeneity, even detecting de novo mutations as disease progresses. In fact, proof-of-concept studies in NSCLC and breast cancer have demonstrated that, although cfDNA analysis does not offer the fidelity of multi-site tissue biopsies, its non-invasive and facile nature can make it a clinically valid tool for real-time monitoring of heterogenous cancer [128,129]. Additionally, methylation-based cfDNA sequencing methods can potentially apply the benefits of liquid biopsy to analyzing the epigenetic development of multiple metastatic lesions as well [130].

Nonetheless, there are a number of drivers of intertumoral heterogeneity that cannot be observed by sequencing alone, even of cfDNA. Non-genetic intertumoral heterogeneity of the TME also plays a prominent role in differential therapeutic outcomes. A pioneering study investigating intertumoral heterogeneity and the immune microenvironment seeded thousands of genetically homogeneous tumors into the ears of mice using a grid of small laser-etched holes [131]. Even in the same host, supposedly identical tumors exhibited very different growth potentials and immune responses, being either immune-inflamed, immune-excluded, or immune-deserted. In immunocompetent mice, a base level of 30% of the tumors were rejected. The immune cell populations of the tumors that formed were similar, but each immune phenotype had distinct T-cell RNA fingerprints.

Some of these immune-oncological variations in the TME may also be deciphered using liquid biopsy; however, a trial that sequenced T-cell receptors (TCRs) from blood-isolated lymphocytes demonstrated that patients with CD8+PD-1+ cells that had high TCR diversity showed greater progression-free survival and response to immunotherapy in NSCLC [132]. This study identified the viability of using TCR sequencing of liquid biopsy-isolated immune cells to provide crucial treatment information. Analysis of blood cytokines, the signaling molecules essential to tumor-immune interactions, can also give clinicians vital diagnostic data. In another NSCLC study, researchers found that interleukin-6 and interferon-γ levels correlated with the quality of a patient’s immune response [133]. By leveraging the advantages of liquid biopsies, such as their non-invasive nature, their potential for real-time analysis, and dynamic monitoring of immune markers across multiple time points, researchers can gain valuable insights that address immune-oncological heterogeneity [134].

**Table 4:** Technologies for assaying intertumoral heterogeneity.

Technology	Principle	Application	Ref.
Blood cytokine analysis	ELISA based quantification of blood cytokines	Characterization of the anti-tumor immune response and inflammation	[133]
cfDNA	Sequencing of DNA isolated from blood	Non-invasive measurement of tumor DNA signature for screening, diagnosis, monitoring, tracking tumor heterogeneity, and directing targeted therapy	[124,127]
cfDNA methylation	Analysis of methylation patterns of tumor cfDNA	Non-invasive characterization of cancer DNA methylation patterns and detection of resistance mechanisms	[130]
TCR sequencing	Sequencing of T-cell receptor genes to profile immune response	Analysis of the immune repertoire by tracking of the anti-tumor immune response through TIL expansion detection	[132]

## 12. Host Heterogeneity

Paired with the increasing accessibility of large data sets, advances in population sciences and computational biology have uncovered a range of characteristics that predispose individuals to or alter the course of cancer. Host heterogeneity encapsulates the diversity between patients, even prior to their developing the disease, specifically referring to the noncancerous biological, genetic, and environmental variables among patients, leading to differential disease progression, treatment response, and overall clinical outcomes (Table 5).

One setting where host heterogeneity is particularly relevant is immune checkpoint blockade (ICB), a therapy that has demonstrated relative success and variable response rates. For PD-L1-targeting therapies, response rates across tumor types are typically up to 50% [135]. In an effort to uncover the disparate efficacy observed across patients, several studies have explored how host heterogeneity affects the response to ICB. Research investigating the role of HLA-I subtypes in ICB response found more robust responses in melanoma patients with heterozygosity in the A, B, and C HLA-I alleles [136]. Additionally, patients with HLA alleles from the B44 family had better overall survival. Other studies have investigated correlations between the gut microbiome and ICB efficacy, suggesting that the presence of the microbe *Akkermansia muciniphila* and an abundance of microbes from the *Ruminococcaceae* predict better outcomes with anti-PD1/PD-L1 therapy in multiple cancer types [137,138]. ICB efficacy also correlates with factors such as weight and age. In a study of 538 metastatic melanoma patients, older patients tended to benefit most from ICB [139]. The researchers successfully reproduced the finding in mice, noting a more suppressed immune presence in the TME in younger mice. With respect to patient weight, obesity has been appreciably linked to immune dysregulation, which plays a potential role in the development of several cancer types [140]. Compellingly, multiple retrospective studies have reported obese patients experiencing more durable ICB responses [141,142]. Mechanistic studies in mice have suggested that this phenomenon may be due to ICB reactivating T-cells that were exhausted as a result of obesity in addition to encouraging cancer cell recognition [143]. In this sense, a cancer that develops in the presence of a dysfunctional immune system is ill-prepared to defend itself from the strong immune response incited by ICB. Sex differences also alter the potential benefit of ICB, with meta-analyses reporting higher overall survival for men treated with anti-CTLA4 therapy and lower hazard ratios for overall survival and progression-free survival with anti-PD-1/PD-L1 therapy [144]. Interestingly, the trend appears to be reversed when ICB and chemotherapy are combined in lung cancer, with women demonstrating significantly more benefit when comparing pooled overall survival hazard ratios [145].

Aside from ICB response, host heterogeneity plays a role in a number of other areas of cancer progression and treatment outcomes. Sex differences have been implicated in different DNA repair processes. For instance, the androgen and estrogen receptors play a role in regulating the DNA damage response, which may consequently cause differences in DNA repair [146]. A recent meta-analysis across different therapeutics and cancer types found that women were more likely to benefit from EGFR inhibitors in non-small cell lung cancer and rituximab in non-Hodgkin’s lymphoma [147]. The same study found that males tend to have fewer side effects during treatment for many classes of therapeutics, causing a potential alteration of the therapeutic window. Another consideration is the host drug metabolism, where genetic polymorphisms in drug-degrading enzymes, such as cytochrome P450s, alter the metabolism of small molecule therapeutic agents [148]. For instance, prodrugs of the anthracycline class can be metabolized along multiple enzymatic pathways. The specific mode of degradation impacts the drug’s efficacy, suggesting that the heterogeneity in degradation enzymes could alter treatment efficacy [149].

Given the influence specific host factors can have on the course of cancer, further research to establish the mechanisms between host variables and observed clinical outcomes can inform treatment or lead to the development of combination therapies to improve patient survival and well-being. With artificial intelligence (AI) models capable of drawing inferences from vast sets of data, the technology has many applications in this space. For one, AI can be used as a predictive model to identify patients most at risk for developing a specific cancer type [150]. AI can also be effectively used to predict the relative efficacy of therapies. For instance, one model integrated patient clinical information with blood measurements and molecular tumor data to forecast immune checkpoint blockade responses [151]. Further technological and computational advances in the fields of population science and epidemiology will continue to elucidate connections between interhost heterogeneity and patient outcomes.

**Table 5:** Technologies for assaying interhost heterogeneity.

Technology	Principle	Application	Ref.
HLA sequencing	DNA sequencing of the human leukocyte antigen (HLA) genes	Characterization, for prognostic and potentially therapeutic purposes, of the relationship between anti-tumor immune response and HLA type	[136]
Drug metabolism phenotyping	Genetic analysis of cytochrome variants affecting drug metabolism	Pharmacogenetic identification of poor and ultra-rapid metabolizers of chemotherapeutics for optimization of anti-neoplastic regimens	[148,149]
Population bioinformatics	Bioinformatic and AI driven synthesis of multi-omics and environmental factors in populations and individuals	Population-level identification of risk factors, prediction of individual disease risk, and prediction of therapy efficacy (e.g., immune checkpoint blockade response rate)	[150,151]

## 13. Conclusions

The variety and breadth with which molecular and clinical heterogeneity manifests itself in cancer can make a comprehensive understanding elusive. Therefore, it was the goal of this article to provide a wide-ranging and expansive survey of heterogeneity in cancer, focusing on equipping the reader with a framework to comprehend and organize the interconnectedness of the various layers of heterogeneity that define this disease. Although we have aimed to elucidate these concepts by featuring the most impactful and illustrative examples in the primary literature, painting a comprehensive picture of cancer heterogeneity is limited by the vastness of this topic. Therefore, a catalogue of first-rate review articles on the individual factettes of cancer heterogeneity has been complied, offering the reader the opportunity for further study (Table 6).

**Figure 2:**
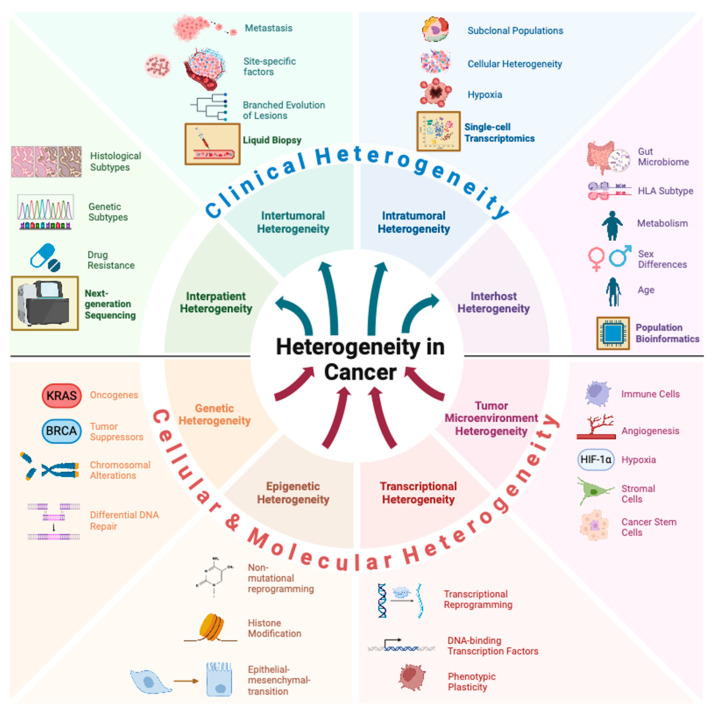
Overview of cancer heterogeneity and emerging technologies.

While we have outlined a plethora of ways heterogeneity manifests in cancer development and progression, it is crucial to identify the forms of heterogeneity that are most clinically relevant (Figure 2). Since metastasis is the cause of death in most patients, gaining an understanding of how heterogeneity relates to the recurrence of metastatic lesions, especially in response to targeted therapies, is essential [1].

A further controversial, yet vital question, is whether, in the light of widespread molecular tumor heterogeneity, single-site biopsies are diagnostically sufficient for providing actionable intelligence on the use of targeted therapies. Multi-site biopsies or single-cell sequencing of tumor samples can allow clinicians to elucidate the heterogeneous features of a patient’s tumor [101]. However, this level of sophistication may be excessive for a surgically resectable lesion. Although multi-site biopsies provide an overview of branch mutations present in a lesion, a single-site biopsy may be sufficient for identifying common truncal driver mutations that are present throughout the primary tumor and therefore in all metastatic lesions. A study investigating the effectiveness of a single primary tumor biopsy in identifying relevant metastatic driver mutations found that across 14 patients any functional driver mutation found in the primary lesion was also found among all metastases [94]. Additionally, the chance that driver mutations found in all metastatic lesions were absent from the primary tumor biopsy was calculated to be below 3%. However, the authors acknowledge that multi-site biopsies may prove valuable in instances of only partially resectable disease, such as glioblastoma, or multifocal lesions, as often observed in esophageal cancer.

In-depth molecular analysis of intra-primary tumor heterogeneity is therapeutically irrelevant in cases where primary tumors can simply be surgically resected, as is the case with around 70% of new cancer cases [152]. In these cases, it is the progression and recurrence of metastatic lesions that are a mortal threat to the patient. Work by Vogelstein and colleagues demonstrated that patients with metastatic lesions demonstrated fairly homogeneous initial responses across all lesions when treated with a targeted therapy that was based on a single tumor biopsy [94]. The problem stems from when these lesions invariably recur due to the existence of genetically, epigenetically, and transcriptionally heterogeneous sub-clones that are resistant. The high analytical resolution of next-generation sequencing (NGS) could allow for the detection of such rare molecular populations. Through a knowledge of known resistance mechanisms and phylogenetic reconstruction, clinicians could discover extant or potentially recurrent subsets of resistant cells and adjust targeted therapies accordingly. Additionally, liquid biopsy analysis of cfDNA allows for the capture of a holistic picture of tumor DNA potentially released by all lesions of sufficient size within a patient. With sufficient sensitivity, this could allow clinicians to understand the heterogeneity of metastatic lesions that are infeasible to biopsy.

While great progress has been made in cancer prevention, diagnosis, and treatment of primary lesions, the prognosis for patients with metastatic disease remains bleak, with only marginal progress being made. This clinical reality necessitates an intensification of efforts to fight metastatic disease. Metastases occur as a result of intratumoral heterogeneity, with cells acquiring capabilities for dissemination via the phenotypic alterations conferred by EMT reprogramming and other described or, as of yet unknown, metastatic signaling pathways [153]. Because only a subset of cells in the primary tumor possess the potential to metastasize, metastases are typically more homogenous than the primary tumor but begin to follow the same clonal evolution after seeding [154]. Cancer stem cells, a rare but integral part of the TME, play a role in enabling metastasis and also contributing to the heterogeneity of metastases [1]. These cells are defined by their ability to initiate lesions and thus could cause recurrence if not fully eradicated.

After seeding, the development of heterogeneity within a metastasis permits differential sub-clonal responses to treatment. Targeting a truncal mutation will create an altered evolutionary environment, granting cells with resistance mechanisms a niche to flourish. A lack of apparent genetic heterogeneity in a metastasis due to its clonal origin may also be deceiving, as the ability to metastasize has potentially given these cells an inherent plasticity and adaptability to new conditions. It follows that phenotypic heterogeneity of genotypically identical cells could be a primary cause of metastatic recurrence.

New transcriptomic technologies will expand the available biomarker data and allow more personalized treatment regimens. AI will also play a role in predicting patient responses and optimizing treatment. The ability to train AI models on various data types allows the technology to be applied in the analysis of complex and inherently heterogenous data sets including NGS, single-cell transcriptomics, and clinical patient data. Take, for instance, a recently published AI approach entitled “PERsonalized Single-Cell Expression-based Planning for Treatments in Oncology (PERCEPTION)” [155]. The strategy implements an AI model trained on single-cell transcriptomic data from cancer cell line drug screens to predict patient responses to targeted agents based on the tumor RNA expression profile. PERCEPTION proved effective in predicting responses to CDK4/6 inhibitors in breast cancer and tyrosine kinase inhibitors in lung cancer. Other groups have trained similar models on gene expression, copy number, and mutation data to predict the efficacy of combination therapies [156]. Machine learning tools like these will enhance the utility of emerging multi-omic technologies. As the predictive power of these algorithms improves clinicians will have powerful tools to draw inference from vast amounts of multi-omic data, facilitating true personalized medicine, at scale. Especially with respect to combination therapy, supporting physicians with AI models may bolster efforts to deliver optimal care, thereby improving patient outcomes.

With this information comes the question of how to optimally treat a disease, and we have yet to see how these predictive algorithms will be implemented. Combination therapies will undoubtedly play a pivotal role in treating heterogeneous metastatic disease but require rigorous assessment for safety. In *EGFR* and *MET* mutant NSCLC, several clinical trials are ongoing to assess combinations of EGFR inhibitors and MET inhibitors [157]. Identifying potentially resistant subpopulations, such as *MET*-amplification in *EGFR*-mutant NSCLC, with emerging technologies would allow for the early targeting of this resistance mechanism through combination regiments.

Understanding and counteracting tumor heterogeneity is paramount for the advancement of cancer treatment, particularly for patients with metastatic disease. The integration of advanced molecular diagnostic technologies aided by powerful computational tools offers a promising avenue to enhance the efficacy of cancer therapies. As we harness these innovations, it is crucial to balance the value of sophisticated diagnostic approaches for research with their clinical practicability. As high-throughput molecular technologies move from the basic science setting into the clinic, it will be increasingly important to understand how to employ these resources to ensure tangible improvements in patient outcomes. Future research should continue to explore the interplay between tumor heterogeneity and treatment resistance, aiming to develop combination therapies that preemptively tackle heterogeneity-driven resistance mechanisms. Combinations of multiple treatment modalities, including targeted therapy, immunotherapy, and chemotherapy, will serve to limit the resistance mechanisms available to tumors, allowing for lasting and potentially curative responses.

**Table 6:** Review articles on individual layers of heterogeneity.

Topic	Author	Title	Year	Ref.
Genetic heterogeneity	Reiter et al.	An analysis of genetic heterogeneity in untreated cancers	2019	[94]
Genetic heterogeneity	Burrell et al.	The causes and consequences of genetic heterogeneity in cancer evolution	2013	[158]
Epigenetic heterogeneity	Sacco et al.	Epithelial–Mesenchymal Plasticity and Epigenetic Heterogeneity in Cancer	2024	[159]
Epigenetic heterogeneity	Carter et al.	The epigenetic basis of cellular heterogeneity	2021	[160]
Intratumoral heterogeneity	Ramon Y Cajal et al.	Clinical implications of intratumor heterogeneity: challenges and opportunities	2020	[161]
Intratumoral heterogeneity	Marusyk et al.	Intra-tumour heterogeneity: a looking glass for cancer?	2012	[162]
Intertumoral heterogeneity	Vogelstein et al.	Cancer Genome Landscapes	2013	[93]
Intertumoral heterogeneity	Buikhuisen et al.	Exploring and modelling colon cancer inter-tumour heterogeneity: opportunities and challenges	2020	[163]
Interhost heterogeneity	Zaal et al.	The Influence of Metabolism on Drug Response in Cancer	2018	[164]
Interhost heterogeneity	Hazini et al.	Deregulation of HLA-I in cancer and its central importance for immunotherapy	2021	[165]

## Data Availability

No new data were created or analyzed in this study. Data sharing is not applicable to this article.
